# Investigating quorum-quenching marine bacilli as potential biocontrol agents for protection of shrimps against Early Mortality Syndrome (EMS)

**DOI:** 10.1038/s41598-023-31197-4

**Published:** 2023-03-12

**Authors:** Moritz Müller, Andrew J. Spiers, Angelica Tan, Aazani Mujahid

**Affiliations:** 1 Faculty of Engineering, Computing and Science, Swinburne University of Technology Sarawak, 93350 Kuching, Malaysia; 2 School of Science, Engineering and Technology, Abertay University, Dundee, DD1 1HG UK; 3 Institute of Biodiversity and Environmental Conservation (IBEC), Universiti Malaysia Sarawak, 93400 Kota Samarahan, Sarawak Malaysia

**Keywords:** Applied microbiology, Biofilms, Industrial microbiology

## Abstract

Early Mortality Syndrome (EMS) has been a major problem for shrimp aquaculture in Southeast Asia due to its epizootic prevalence within the region since the first reported case in 2009. This study explores the application of halophilic marine bacilli isolated from coral mucus and their quorum-quenching abilities as potential biocontrol agents in aquaculture systems to combat the causative agent of EMS, *Vibrio parahaemolyticus*. *N*-acylhomoserine lactone (AHL)-degrading (AiiA) activity was first screened by PCR then confirmed by bio-reporter assay, and a combination of 16S rDNA sequence analysis and quantitative phenotype assays including biofilm-formation and temperature-growth responses were used to demonstrate diversity amongst these quorum-quenching isolates. Three phenotypically distinct strains showing notable potential were chosen to undergo co-cultivation as a method for strain improvement via long term exposure to the pathogenic *V. parahaemolyticus*. The novel approach taken led to significant improvements in antagonism and quorum quenching activities as compared to the ancestral wild-type strains and offers a potential solution as well as pathway to improve existing beneficial microbes for one of the most pressing issues in shrimp aquacultures worldwide.

## Introduction

The production of seafood has undergone a dramatic transition since the 1980s to include fish farming or aquaculture. One of the most lucrative and widely traded aquaculture products are shrimps, with the commercial sale of shrimp a substantial source of revenue for many developing countries and Asia producing almost 90% of all aquaculture products worldwide^1–3^. China is the world's largest aquaculture producer and alone produces 50 percent of penaid shrimps globally^4^, with tiger shrimp (*Penaeus monodon*) and Pacific white shrimp (*Penaeus vannamei*) among the common cultured species^5,6^. Persistent diseases and related issues have, however, reduced the production of farmed shrimp in China and Vietnam^1^ as the nature of intensive shrimp farming makes shrimps highly susceptible to diseases such as Early Mortality Syndrome (EMS; or Acute Hepatopancreatic Necrotic Syndrome, AHPNS) which occurs within the first 35 days after stocking cultivation ponds^7^. EMS is caused by *Vibrio parahaemolyticus* which can also produce gastroenteritis in people through poorly prepared seafood and contaminated water^8,9^. *V. parahaemolyticus* utilises quorum sensing (QS) to regulate the expression of virulence factors including the production of toxins and biofilm-formation.

Bacterial communication systems depend on autoinducers (AIs) as signalling molecules. Three major classes of AIs are known: *N*-acyl Homoserine Lactones (AHLs) or Autoinducer-1, Autoinducing Peptides (AIPs) and Autoinducer-2 (AI-2)^10^. QS systems are widespread amongst *Vibrio* spp.; addition of the signalling molecule C6-HSL to bacterial media, for example, led to an increase in biofilm formation by 29%^11^. They act through complex signal transduction pathways involving the synthesis and detection of N-acylhomoserine lactone (AHL) signal molecules known as autoinducers which lead to the expression of virulence factors only when high cell densities are achieved^12^.

QS-regulated virulence is often modulated by exogenous AHL-degrading enzymes known as auto-inducer inactivation proteins (AiiA) or AHL-lactonases^13,14^. Subsequently, several bacteria have been developed as quorum-quenching (QQ) biocontrol agents that inhibit a range of pathogenic bacteria in farmed or aquarium fish^15–18^. Recent studies of potential bio-control bacilli have focussed on their colonisation abilities in aquaculture systems, and commonly advocate the use of strains isolated from sources similar to the tank conditions^19^ as these would not require further acclimatisation (i.e. genetic adaptation) to new temperature, salinity, or oxygen regimes.

This research, however, looks into a novel source of potential biocontrol agents based on the Coral Probiotic Hypothesis which seeks to explain coral resistance to pathogens through selection for a community of microorganisms including the coral (the holobiont) which reduces the stress experienced by the coral^20^. In particular, mucus-associated bacteria provide a physicochemical barrier between the external environment and the rest of the coral holobiont which also protects against pathogen invasion^21–25^. Here we identify coral mucus-associated bacilli with QQ activity as potential biocontrol agents which might be used against *V. parahaemolyticus* in shrimp aquaculture systems. We explore the diversity of colonisation-associated abilities amongst these isolates and demonstrate that *Bacilli*-*Vibrio* co-cultivation could be used for further strain improvement. This type of ecological-evolutionary approach may provide a long term solution to EMS/AHPNS with less likelihood of *V. parahaemolyticus* strains developing resistance in aquaculture systems in the future.

## Results

### Primary screening of quorum quenching bacilli isolated from coral mucus

A total of 55 coral mucus bacilli were isolated from coral mucus samples collected in the Talang Satang reef^26^. Of these, 30 showed some evidence of an antagonistic effect reducing *Vibrio parahaemolyticus* growth and a potential QQ effect reducing the quorum regulated C6-HSL-mediated production of pigment by *Chromobacterium violaceum* in plate-based assays. We then screened these strains for *Bacillus* spp. AHL-lactonase-like sequences by PCR amplification of genomic DNA after Pan et al.^27^ and obtained strong single-amplicon signals for 14 strains and weaker responses for a further six strains.

**Figure 1:**
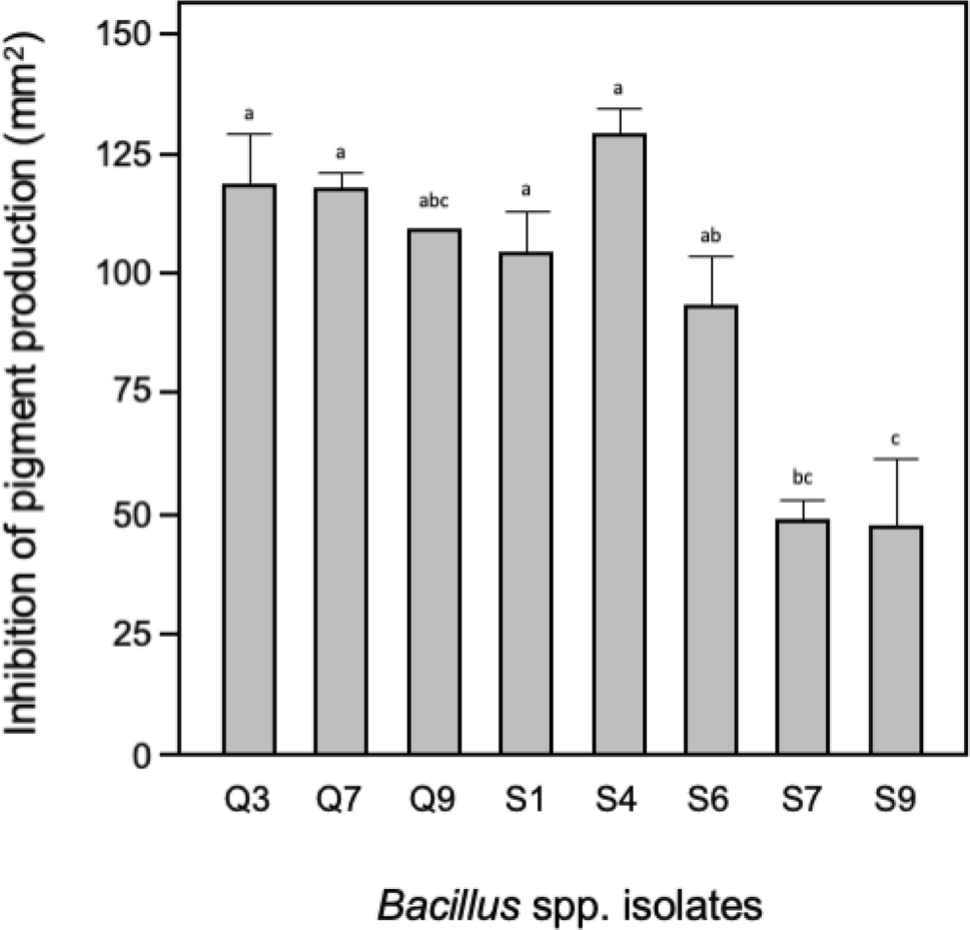
QQ graph. Isolates are shown on the x-axis. Means ± SE are shown on the y-axis; means not connected by the same letter are significantly different (TK-HSD, α = 0.05).

Of those strains producing a strong PCR signal, at least eight strains were found to be able to grow on minimal plates containing the short-chain AHL C6-HSL as the sole carbon source^28^ demonstrating that they were capable of expressing functional AHL-lactonases under the conditions used here. Soluble AHL-lactonase activity in cell-free culture filtrates was further confirmed using a modified well-diffusion assay in which *C. violaceum* pigment production is effected by QQ activity which reduces C6-HSL levels^29–31^. Significant differences in activity were found (ANOVA, p < 0.05) with strains Q3, Q7, Q9 and S4 producing the greatest QQ activity under the conditions tested here (Fig. 1 and Table 1). We then further characterised these eight strains in order to identify potential QQ biocontrol agents which could be used to colonise aquaculture systems or even shrimp digestive tracts to prevent or reduce the establishment of pathogenic *Vibrio* populations leading to EMS and shrimp mortality.

**Table 1:** Characterisation of Bacilli strain colonisation-associated behaviours.

Bacilli	AHL-Lactonase activity^1^	Swimming motility^2^	Liquid surface tension^3^	Relative surface hydrophobicity^4^	Static microcosm growth^5,6^	Biofilm attachment^5,7^	Biofilm strength^5,8^	HCA cluster^9^	Qualitative biofilm phenotype^10^
Q3	118 ± 8^a^	36.8 ± 0.6^a^	54.55 ± 0.72^a^	1.287 ± 0.016^a^	1.073 ± 0.081^a^	0.282 ± 0.026^a^	0.042 ± 0.008^a^	1	PC/PC/PC/WA/–
Q7	118 ± 9^a^	29.3 ± 0.7^c^	53.95 ± 0.92^a^*	0.993 ± 0.007^b^	1.961 ± 0.021^a^	0.876 ± 0.037^a^	0.013 ± 0.001^a^	2	–/VM/FM/FM/–
Q9	109 ± 16^abc^	33.7 ± 0.7^ab^	52.30 ± 0.61^a^*	0.946 ± 0.014^b^	1.914 ± 0.017^a^	1.202 ± 0.060^a^	0.006 ± 0.002^a^	2	–/VM/–/–/–
S1	104 ± 9^a^	30.5 ± 1.3^bc^	53.46 ± 0.70^a^*	0.965 ± 0.012^b^	1.940 ± 0.062^a^	2.534 ± 0.117^a^	0.022 ± 0.006^a^	3	FM/PC/VM/VM/–
S4	129 ± 9^a^	27.7 ± 0.9^ cd^	55.81 ± 1.72^a^	0.626 ± 0.022^c^	1.922 ± 0.013^a^	2.021 ± 0.076^a^	0.0115 ± 0.000^a^	4	FM/VM/FM/–/–
S6	93 ± 8^ab^	24.3 ± 0.9^d^	47.82 ± 0.74^b^*	0.934 ± 0.007^b^	1.769 ± 0.016^a^	2.053 ± 0.069^a^	0.007 ± 0.002^a^	4	–/PC/VM/–/–
S7	49 ± 11^bc^	27.3 ± 0.4^ cd^	51.91 ± 0.26^ab^*	0.968 ± 0.008^b^	2.184 ± 0.018^a^	1.928 ± 0.075^a^	0.010 ± 0.001^a^	3	–/FM/FM/–/–
S9	48 ± 11^c^	29.5 ± 0.5^c^	54.02 ± 0.13^a^*	0.984 ± 0.010^b^	1.803 ± 0.023^a^	2.522 ± 0.053^a^	0.010 ± 0.001^a^	3	–/PC/VM/–/–

**Figure 2:**
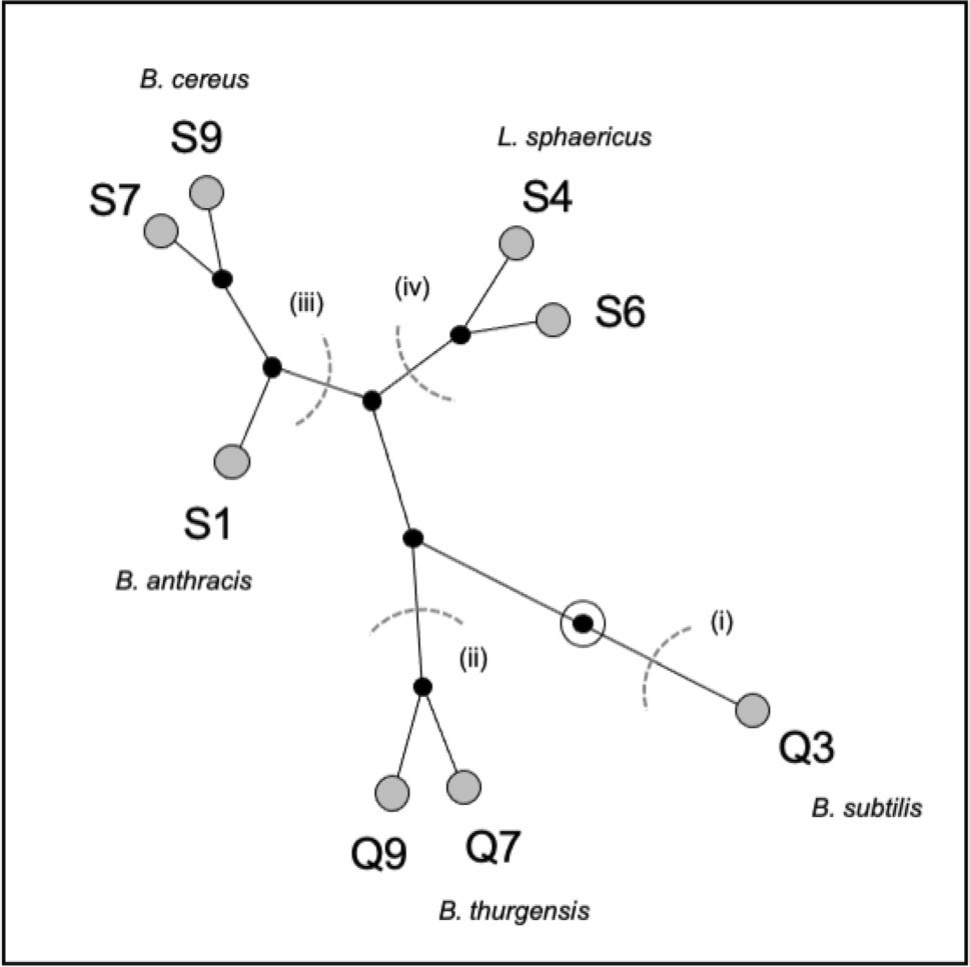
Shown here is a HCA constellation plot which clusters similar strains in terminal (short) branches and links strains with greater differences with longer branches. The plot is arbitrarily rooted mid-way along the longest branch (circled) and the four major groups (grey arcs) determined automatically. Those strains that have been identified by 16S rDNA analysis are indicated.

**1,** AHL Lactonase activity measured as mm^2^ inhibition/24 h. **2,** Swimming measured as mm/24 h. **3,** Liquid surface tension determined for static KB cultures as mN/m after 3 days incubation (the liquid surface tension of sterile KB was 57.62 ± 0.09 mN/m). **4,** Measured by relative differences in OD_600_. **5,** From combined biofilm assays using KB microcosms. **6,** Growth measured as final population size, OD_600_. **7,** Biofilm attachment to the vial walls measured using Crystal violet, A_570_. **8,** Biofilm strength measure using glass balls, g. **9,** From the HCA Constellation plot shown in Fig. 2. **10,** Biofilm-formation was tested in LB, KB, BHI, NB and M9-Gluc microcosms (left to right) and categorised as FM (Floccular Mass) PC (Physically Cohesive), VM (Viscous Mass) or WA (Waxy Aggregate) class biofilms (–, no biofilm observed). Means and SE are shown; means with same superscript letter are not significantly different (within assays, TK-HSD, α = 0.05). *, Significantly different from sterile medium control (Dunnett’s, p < 0.05).

### Characterizing the colonisation abilities of the quorum quenching bacilli

We subjected the eight QQ bacilli to a series of simple assays focussing on key abilities relevant to the colonisation of aquaculture systems, including swimming motility, the ability to modify liquid surface tension and surface conditions through possible surfactant production, and relative cell surface hydrophobicity, which affect the ability of bacterial cells to approach, modify and settle on appropriate surfaces, as well as growth rates, attachment levels, and biofilm-formation necessary for long-term colonisation (Table 1). Significant phenotypic variation was found between strains in these assays (ANOVA, p < 0.05) which is summarised in a Hierarchical cluster analysis (HCA) constellation plot in which strains are grouped according to similarity (Fig. 2) (the successful differentiation of all strains from one another in this analysis also demonstrates that we had not isolated the same strain more than once). This variation was also reflected in the diversity of 16S rDNA sequences we obtained for several strains and we have tentatively identify strain Q3 as *Bacillus subtilis,* Q7 as *Bacillus thuringiensis,* S1 as *Bacillus anthracis,* S4 as *Lysinibacillus sphaericus,* and S9 as *Bacillus cereus,* though we note that there is no direct mapping between our phenotype clustering and species phylogenies. Given the extensive diversity within the *Bacillus* and sister genera which include the *Lysinibacillus*, it is not surprising that the closest 16S rDNA homologues to our coral mucus strains are from the better characterised soil-associated bacilli and in each of these species AHL-lactonase gene sequences have already been identified^32^.

**Figure 3:**
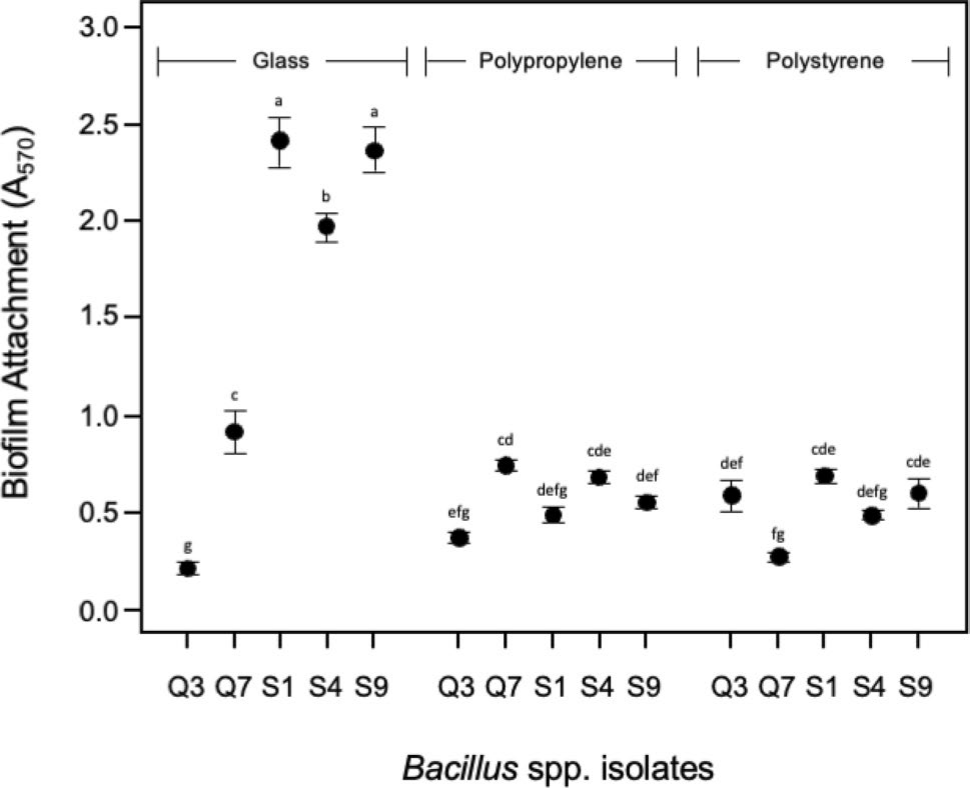
Attachment to different surfaces in KB. Attachment assay after 24 h. Means ± SE are shown on the y-axis and isolates on the x-axis. Means not connected by the same letter are significantly different (TK-HSD, α = 0.05).

**Figure 4:**
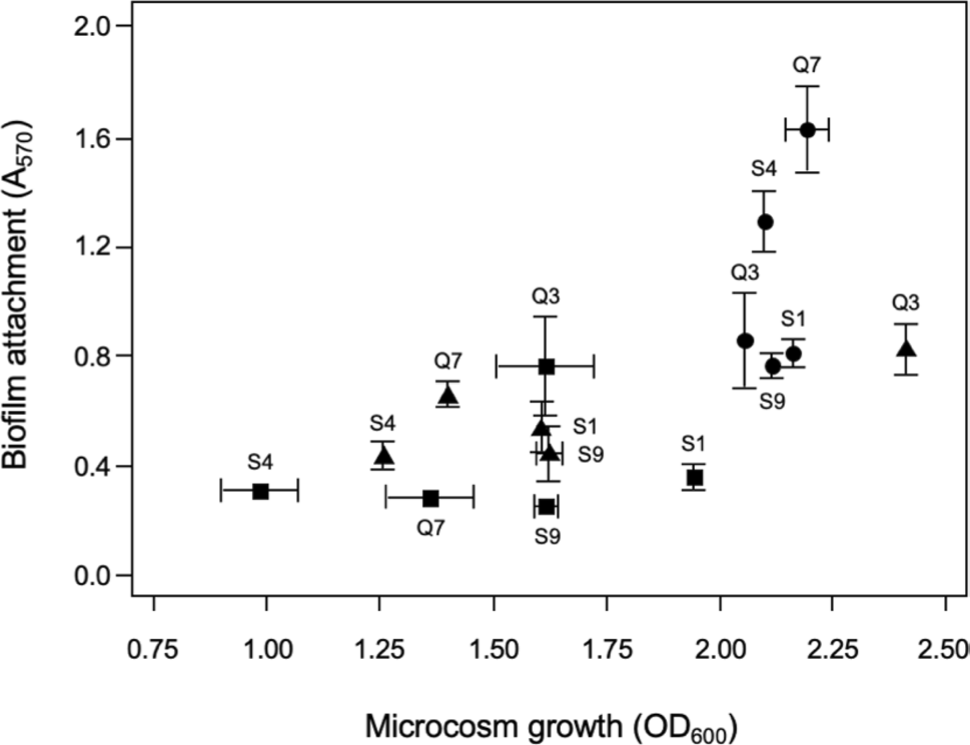
Combined biofilm assay after 3 days incubation. KB, circle, LB square, BHI, triangle. Biofilm attachment/growth in different media. Means ± SE are shown; significant differences between biofilm attachment levels (A_570_) and microcosm growth (OD_600_) were found between strains (ANOVA, p < 0.05) but for the sake of clarity these are not shown in this figure.

In our preliminary characterisation of biofilm-formation in static microcosms after Ude et al*.*^33^ we noted considerable variation seen in the qualitative phenotypes produced when bacilli were tested with different growth media (Table 1). As biofilm-formation is likely to be a critical behaviour allowing the colonisation of aquaculture tanks and possibly also shrimp exoskeleton and intestinal mucosa, we investigated surface attachment and biofilm-formation more closely using five strains (Q3, Q7, S1, S4 and S9) chosen to represent the phenotype diversity seen within this set of strains and shown in Fig. 2. All strains were capable of swimming motility (Table 1) indicating that cells could approach submerged solid surfaces. However, although significant differences in relative attachment to glass, polypropylene and polystyrene were observed between strains (ANOVA, p < 0.05) (Fig. 3), no significant pair-wise correlations were found between attachment levels on different surfaces (p < 0.05). Strains Q3 and S4 with significantly different relative (cell) surface hydrophobicities (Table 1) show different attachment behaviour to glass surfaces but not to polypropylene or polystyrene. It is unlikely that any strain is modifying surface conditions through the expression of surfactants as none were able to reduce the liquid surface tension in static KB cultures (Table 1). The attachment assays suggest that each strain was sensing each surface differently which subsequently affected attachment levels and early micro-colony formation. Similarly, a comparison of biofilm-formation in BHI, KB and LB microcosms showed significant variation between strains in terms of growth and biofilm attachment levels (ANOVA, p < 0.05), and in BHI microcosms only strain Q3 produced biofilms with a significantly higher strength (TK-HSD, α = 0.05) (Fig. 4). We investigated this further taking a General Linear Modelling (GLM) approach and found that *strain*, *media* and *strain x media* interactions were all significant effects on growth, biofilm attachment levels and strength (ANOVA, p < 0.0001), and confirm that BHI, KB and LB had differential effects on growth and attachment levels (LSMeans difference Tukey-HSD, α = 0.05). These findings are further evidence that there is significant behavioural differences amongst these strains which might impact on their ability to colonise surfaces in aquaculture tanks or shrimp.

**Figure 5:**
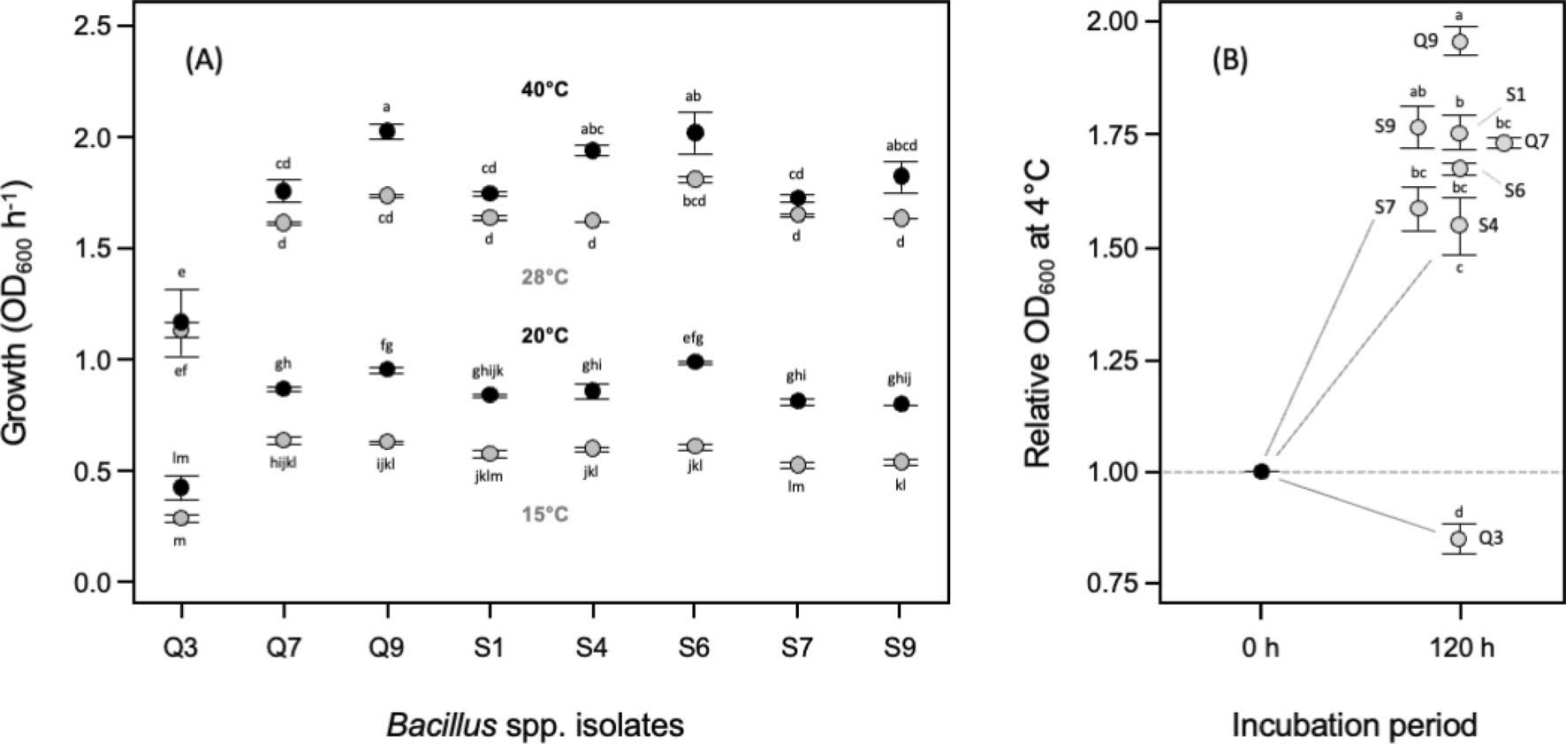
In KB. (**A**) Temperature response (15–40 °C/24 h) and (**B**) growth/survival at 4.5 °C/120 h). Means ± SE are shown on the y-axis and isolates on the x-axis. Means not connected by the same letter are significantly different (TK-HSD, α = 0.05).

Temperature is another key parameter for microbial growth and we observed significant differences between strains, most notably Q3 which grew very poorly compared to all others. A GLM highlighted the significant effects of *strain*, *temperature* and *strain × temperature* (Fig. 5A). In terms of practical application in the field, it is important to note that all assays were conducted at 20–22 °C which represents conditions similar to the shrimp aquaculture in Asia. Food trade is a global business and seafood such as shrimp is often transported significant distances on ice, and during this time pathogen populations may continue to develop. To assess the potential of our bacilli isolates to keep suppressing the pathogen, we also looked at differences in survival or growth of the QQ bacilli at 4 °C—(Fig. 5B). Q3 did not perform well but all other strains did, again highlighting the differences across the strains but also, more importantly, their potential to be used as biocontrol at different stages of the production.

### Potential for strain improvement by co-culturing with *V. parahaemolyticus*

**Figure 6:**
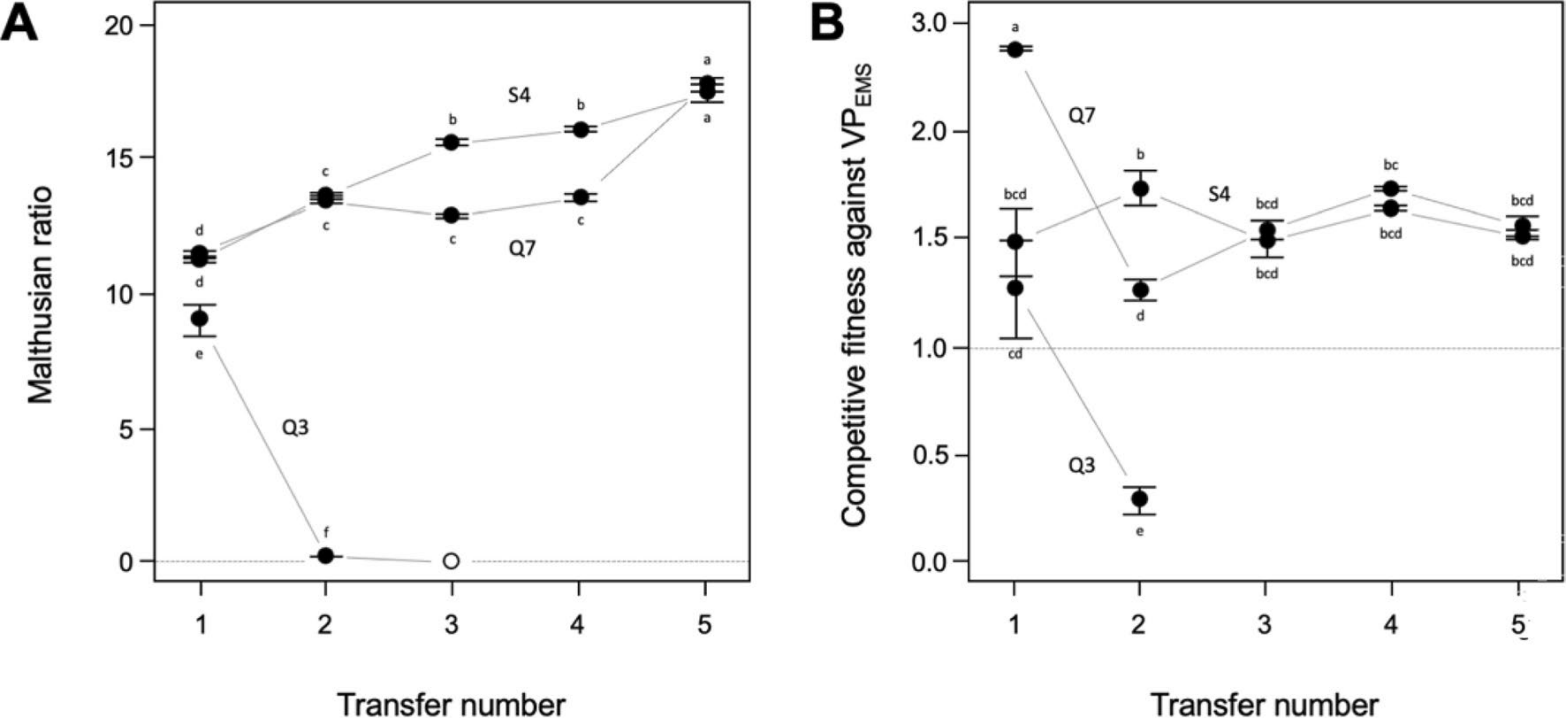
(**A**) Static—Malthusian ratio (no VP_EMS_) and (**B**) competitive fitness against VP_EMS_.

As described above, three strains (Q3, Q7, and S4) stood out in terms of their potential use as probiotic and were chosen for an additional co-cultivation to further improve their capacities. Q7 and S4 showed strong growth improvement across transfers, whereas Q3 did not grow after the second transfer anymore (Fig. 6a). The latter was also reflected in a loss of competitive fitness of Q3 against VP_EMS_ (Fig. 6b). While the exact cause for Q3’s sudden drop in performance warrants further investigation, it is clear that further coevolution work on Q3 would be redundant based on the observed erratic in vitro behaviour. Competitive fitness across the transfers for strains Q7 and S4 were significantly different compared to Q3 (Tukey–Kramer HSD, q* = 2.935, α = 0.05). Both Q7 and S4 co-cultured strains showed significant advantages over VP_EMS_ regardless of the conditions in which they were grown together (p < 0.0001). Q7, however, loose in competitive fitness, indicating a need to replenish its dose if used as bio-control.

Growth under shaking conditions (both aerobic and microaerobic) was not significantly changed for any strain. Coevolution under static conditions, on the other hand, enabled co-cultured strains to grow better than wild types in microaerobic condition (ANOVA, p = 0.0471) and comparable to that of VP_EMS_ (|d|= 2.890, α = 0.05). This is a significant finding as it suggests that the bacilli strains, commonly described as aerobic bacteria, have gained enhanced ability to survive in microaerobic conditions, and are hence able to compete with VP_EMS_ which are well known facultative anaerobes.

**Figure 7:**
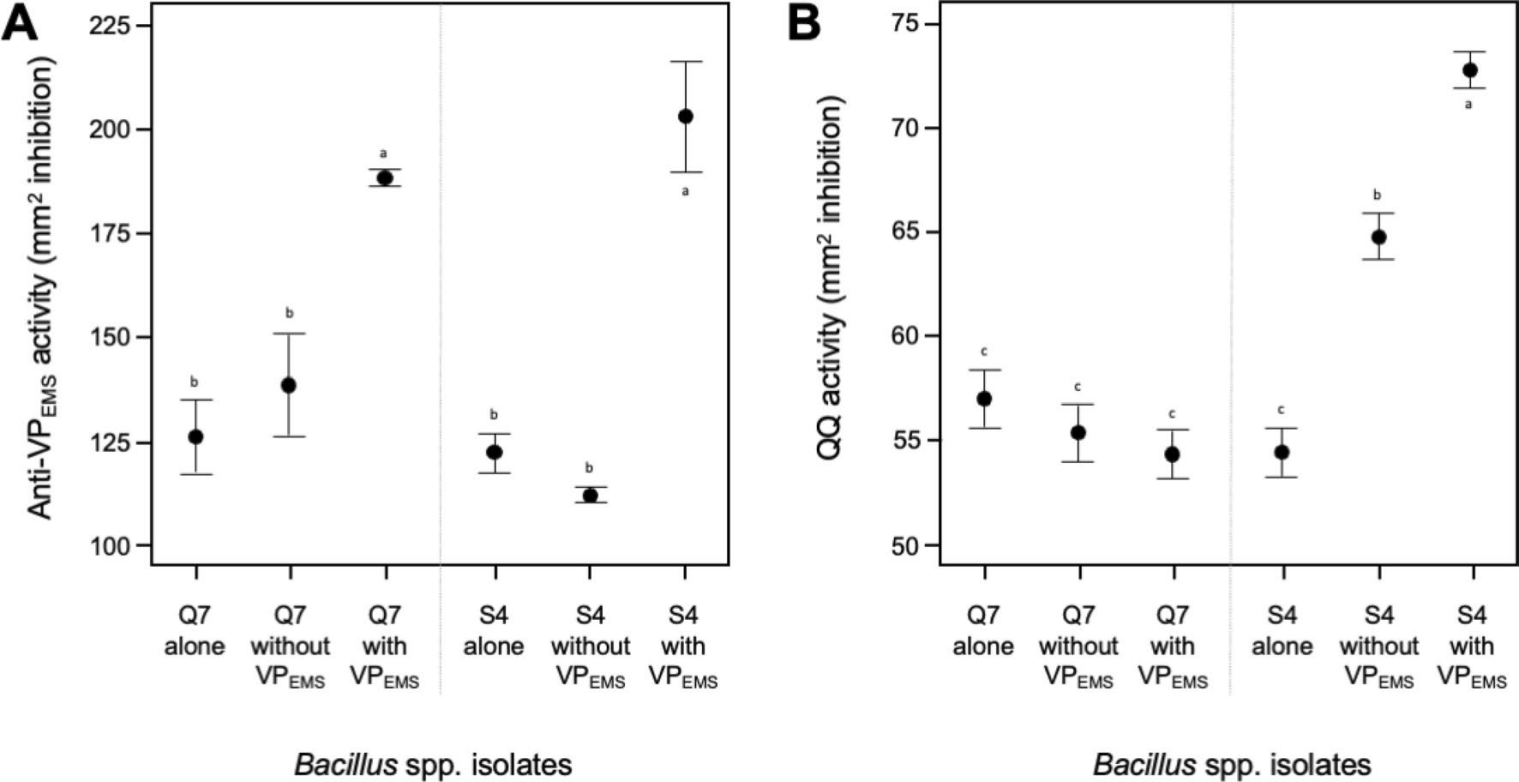
(**A**) Anti-VP_EMS_ activity (in mm^2^ inhibition) and (**B**) quorum quenching activity (in mm^2^ inhibition) for both Q7 and S4.

Supporting their potential use as probiotic, strains Q7 and S4 displayed significant fitness advantages over VP_EMS_ (*P* < 0.05); regardless of the conditions in which they were grown together (*P* < 0.0001; Fig. 7). Significant inhibition of VP_EMS_ was observed by successfully co-cultivated strains Q7 (*P* < 0.0001) and S4 (*P* = 0.0002). Comparison of inhibitory effects when wild type was used is shown in Fig. 7A, proving that co-cultivation did bring about enhancement in VP_EMS_ antibiosis. Both Q7 wild type and co-cultivated strain showed no significant difference in their quorum quenching abilities (t-test, P = 0.150). However, co-cultivated S4 has improved quorum quenching abilities as compared to its wild type. A t-test conducted confirmed significant improvement in quorum quenching abilities (P < 0.0001) for co-cultivated S4 (Fig. 7B).

## Discussion

Current methods used by the shrimp aquaculture industry for control and prevention of EMS involve direct chemotherapy using competitive inhibitors of the aromatic class to disrupt extracellular quorum signalling between *Vibrio parahaemolyticus* cells or excess production of AHL-lactonases via heterologous gene expression at an industrial scale^34–39^. These direct methods of inhibiting quorum sensing could potentially lead to pathogenic *V. parahaemolyticus* adapting against these compounds. There will always be a risk of emergence of resistant populations of pathogens which could easily render current generations of anti-quorum sensing compounds ineffective.

As the concentration of AHLs is a factor in virulence gene expression in pathogenic bacteria, it is possible to modulate the availability of AHLs as signalling molecules in order for prevention of pathogenicity. Several studies looked into AHL-degrading enzymes, broadly categorised as autoinducer inactivation proteins (AiiA)^27,40,41^ as a tool for overcoming interspecies competition^42^. Bacilli have been developed as biocontrol agents for shark catfish^18^, catfish^19^, tilapia^20^ and carp^33^. Studies have shown bacilli are common gut microbiota of healthy shrimp and many have been suggested as potential probiotics^15,43–46^.

The ideal probiont for an aquaculture system would be genetically manipulatable but stable and easily controlled when in use^47^. Such probionts would modify pathogen loads by competitive exclusion and immunomodulation within the animal host. Naturally occurring coevolution is typically viewed as a problem due to continual adaptations of pathogens to better infect hosts. One can, however, also apply it to generate a superior (fitter) organism adapted to biotic stress^48^ and/or increase the production of bioactive compounds^49^. We designed this study on the premise that wild type bacilli strains which possess desired traits would increase their activity upon repetitive exposure to a VP_EMS_ strain to the point that a significant improvement between co-cultivated and wild type strains can be observed. Previous coevolution studies focused on host–pathogen interactions^50–53^ and expression of bacterial toxins^54^, however, we are not aware of any body of work detailing the use of co-cultivation in the context of shrimp or other aquaculture diseases.

Co-cultivation as a method for strain improvement allows for reciprocal co-evolution of the selected biocontrol agents against the pathogenic wild type *V. parahaemolyticus*. Therefore, this method may provide a long term solution to EMS with less likelihood of VP_EMS_ developing resistance. Assessment of the strains’ phenotypic characterization whilst simulating *in-situ* conditions showed favourable growths for Q7 and S4, which is expected of promising probionts. Q7 and S4 were able to survive both low and elevated temperatures, grew well in saline environment, and are motile. Air–liquid interface biofilms of both strains were found to have good attachment levels on inorganic surfaces. The bacilli responded well to the high-nutrient (nitrogen) content in KB but experienced slightly retarded growth due to the exogenous Fe. This relationship between media and enhanced growth of probiotic bacteria led us to postulate that shrimp feed content should contain high nitrogen but low Fe levels as a crucial prebiotic factor.

Co-cultured strains possessed several key advantages over their wild types. They showed significantly increased QQ activity, increased growth rates, and importantly gained enhanced ability to survive in microaerobic conditions. The latter enables them to survive and grow (at the same rates) in oxygen deficient shrimp gastrointestinal tract and hence compete with VP_EMS_. Our approach can be used to improve other probionts as well without compromising the host’s immune response, increasing the applicability of antivirulence therapies and reducing the necessity of antibiotics, ultimately leading to safer and more sustainable seafood.

## Conclusion

This study highlighted the potential of selected bacilli strains from a unique marine source as biocontrol agents in shrimp aquaculture. Several bacilli strains from coral mucus were able to quorum quench communication molecules of the known shrimp pathogen* Vibrio parahaemolyticus *in vitro, and deter it from reaching threshold for pathogenicity activation. Further in vitro assessments, following co-cultivation resulted in S4, related to *Lysinibacillus sphaericus*, being selected as the most promising candidate for further in situ and in vivo studies. Further work needs be carried out to assess other autoinducers such as AIPs and also other AHLs. Importantly, further work also needs to be carried out for the purpose of in-situ applications as this strain could enable sustainable shrimp farming without the over-reliance on antibiotics as a disease control method.

## Methods

### Isolation and culturing of bacilli

Corals were inverted to allow mucus layers to drip for collection and bacteria isolated as previously described^26^. In short, mucus samples of 50 µL were spread evenly onto Zobell marine agar plates (HiMedia, Malaysia) and allowed to dry for 10 min before UV-sterilization (320 nm) for 10 min to provide a substrate for bacterial growth. Another 50 µL layer of mucus was applied without sterilization and the plates incubated for 24 h at 30 °C. Bacilli were then plated onto selective HiCrome *Bacillus* agar (HiMedia, Malaysia) and gram-stained to confirm identity before further analyses.

Bacilli were incubated in a range of different media to assess phenotypes and behaviours. These included King’s B (KB^55^), Luria Bertani (LB; HiMedia, Malaysia), Brain Heart Infusion (BHI) and Nutrient Broth (NB; Oxoid, UK), minimal M9 salts with glucose (M9-Gluc; NaCl 1.0 g/L, KCl 0.5 g/L, MgSO_4_ 0.05 g/L, CaCl_2_ 0.06 g/L, KH_2_PO_4_ 0.2 g/L, c glucose 20 mM, final pH 7.5), and minimal sea salt media with C6-HSL (MSS-C6-HSL; NaCl 1.0 g/L, KCl 0.5 g/L, MgCl∙6H_2_O 0.4 g/L, CaCl_2_∙2H_2_O 0.1 g/L, KH_2_PO_4_ 0.2 g/L, Na_2_SO_4_ 0.15 g/L, MES 1.0 g/L, artificial sea salt 0.01 × 10^–9^ g/L (Sigma-Aldrich, Malaysia), C6-HSL 24 nM, final pH of 7.5). Agar at 1.2% (w/v) was added for plates. Microcosms were 30 mL lidded glass universal vials containing 6 ml liquid media and were incubated with shaking or without (static). Other bacteria were regularly cultured in LB at 28–30 °C when required.

### Preliminary screening of bacilli using antagonistic and QQ plate-based assays

A virulent strain of *Vibrio parahaemolyticus* (abbreviated in this study as VP_EMS_) which had been obtained from a local shrimp company (Sea Horse Corporation, Kuching, Malaysia) was used for plate-based antagonistic assays^31^. Over-night LB cultures of bacilli and VP_EMS_ were used to drop (25 µL)-inoculate modified TSA plates containing pancreatic digest of casein 15.0 g/L, papaic digest of soyabean meal 5.0 g/L, and sodium chloride 20.0 g/L (n = 3) which were incubated at 30 °C for 48 h to assess potential antagonistic effects reducing VP_EMS_ growth. Bacilli and *Chromobacterium violaceum* DSM 30191 (German Collection of Microorganisms and Cell Cultures GmbH, Germany) over-night LB cultures were also streaked in parallel rows onto modified TSA plates which were incubated for 24 h to assess potential QQ effects reducing normal *C. violaceum* violet pigment production which is regulated by C6-HSL^31^.

### 16S rDNA identification and determining the presence of AHL-lactonase sequences

Bacilli genomic DNA was extraction using PureLink Pro 96 Genomic DNA Purification Kit (Thermo Fisher Scientific, Malaysia) and the 16S rRNA region amplified by PCR using Red Taq (Bioline, Malaysia) and 8F/519R primer pairs^56^. These amplicons were then sequenced by the Beijing Genomics Institute (Beijing, China) and analysed using Basic Local Alignment Search Tool software (National Center for Biotechnology Information, USA) to identify strains to the species level. The AHL-lactonase (*aiiA*) gene was similarly amplified using using AiiA1 and AiiA2 primers^27^.

### Quantitative measurement of quorum quenching (QQ) activity

QQ activity was assessed by bio-reporter assay using *C. violaceum* DSM 30191 in a modified well-diffusion assay^29–31^. Bacilli were incubated in MSS-C6-HSL for 24 h, and cell-free filtrates produced by passage through a 0.22 µm membrane filter to provide a source of AiiA^28^. LB plates were spread with 300 µL of over-night *C. violaceum* LB culture (n = 8) and dried for 5 min. A 5 mm diameter plug was removed from the centre of each plate which was then filled with 20 µL bacilli filtrate. The plates were incubated at 30 °C for 24–72 h before photography and the *C. violaceum* non-pigmented (inhibitory) zone corresponding to the QQ activity was measured using ImageJ^57^. An assumption made using this assay is that the QQ activity is only affected by the amount and activity of AHL-lactonase present in the bacilli filtrates.

### Other quantitative phenotypes used to differentiate bacilli strains

Quantitative characterization of bacilli was undertaken using swimming, cell hydrophobicity, surface tension, biofilm and temperature-growth assays. Swimming motility was assessed using soft-agar KB plates containing 0.1 × normal nutrient levels and 0.3% (w/v) agar. Aliquots of 10 µL of over-night KB cultures (n = 8) were stabbed into the agar and the diameter of the bacterial expansion measured (mm) after 24 h. Relative cell hydrophobicity (*H*r) was determined by the microbial adhesion to hydrocarbons (MATH) assay with hexadecane^58^ using over-night KB cultures (n = 3). Cell-free 72 h KB culture supernatants (n = 3) were used to determine liquid surface tension (mN/m) at 20–22 °C using a K100 Mk 2 Tensiometer (Krüss GmbH, Germany) after Koza et al*.*^59^ (the liquid surface tension of deionised water was 73.31 ± 0.13 mN/m and sterile KB 57.62 ± 0.09 mN/m). Biofilm-formation at the air–liquid (A–L) interface in static microcosms which were inoculated with 100 µL of over-night culture and incubated statically for 7 days at 20–22 °C before inspection and categorization of biofilm types^33^. Biofilm-formation was quantified using the combined biofilm assay^60^ with microcosms (n = 8) sequentially assayed for biofilm strength (grams) using glass balls, attachment levels to the vial walls measured using Crystal violet staining (A_570_), and total microcosm growth determined from optical density (OD_600_) measurements after 3 days. Temperature-growth profiles in KB microcosms (n = 3) were determined by OD_600_ measurements incubated at 15–40 °C for 24 h. Similarly, survival at 4 °C was assessed over 5 days but data is expressed as relative growth compared to time zero.

### Co-cultivation of bacilli and fitness measurements

A combined co-cultivation and competitive fitness experiment, modified from Charusanti et al.^48^ and Koza et al.^59^, was undertaken to assess whether bacilli could better adapt to growing in the presence of VP_EMS_ in static microcosms over a period of 24 days. A 1:1 mixture of over-night bacilli and VP_EMS_ cultures was prepared and 100 µL used to inoculate two sets of replicate (n = 3) KB microcosms, one set incubated statically and the other with shaking at 20–22 °C for 4 days. Serial dilutions of the initial 1:1 mixture and the co-cultivated populations sampled from the microcosms after vigorous mixing were prepared and spread onto a differential media (DM; tryptone (5.0 g/L), proteose peptone 5.0 g/L, yeast extract 1.0 g/L, sucrose 10.0 g/L, NaCl 50.0 g/L, phenol red 0.025 g/L, and bacteriological agar 17.0 g/L, final pH of 8.2) to enumerate bacilli and VP_EMS_ colonies. Individual bacilli colonies (n = 20) were randomly chosen and samples taken using sterile loop, combined and re-suspended in 2.0 mL of KB broth to be used as inoculum. Subsequent periods of incubation were carried out by inoculating fresh KB microcosms (6.0 mL) with equal volumes of bacilli mixture (50 µL) and wild type VP_EMS_ culture (50 µL). In selecting the best growth medium for co-cultivation studies, KB and KB supplemented with 100 µM Fe_2_(SO4)_3_ were chosen. Populations were incubated in microcosms (n = 6) over a six week period in which cultures were incubated for a total of 24 days. Bacilli competitive fitness (W) was determined for each transfer as the ratio of the Malthusian parameters^61^ as *ln* [*Bacillus*_final_/*Bacillus*_initial_]/*ln* [VP_final_/VP_initial_]. A similar transfer experiment was undertaken with just bacilli as a control, and adaptation to KB media recorded as *ln* [*Bacillus*_final_/*Bacillus*_initial_].

### Statistical analyses and modelling

All assays were conducted with replicates, and means and standard errors (SE) are provided where necessary. JMP Statistical Discovery Software v12 (SAS Institute Inc., USA), SPSS Statistics 22.0 (IBM, USA) and Microsoft Excel 2010 (Microsoft, USA) were used to analyse data. Differences between means were tested by ANOVA and post hoc multiple comparison tests including Dunnett’s method with a control and Tukey–Kramer HSD (TK-HSD). Hierarchical cluster analysis (HCA) using the Ward method with equal weighting between factors was used to investigate similarities between strains. A general linear modelling (GLM) approach with significant effects examined by LSMeans Differences Tukey HSD tests was also used to examine data sets. T-tests (*t*) were used to determine whether competitive fitness was significantly different to one (W ≠ 1).

## Data Availability

The sequences associated with this manuscript have been deposited at the NCBI GenBank under accession numbers ON197135-ON197138.
